# Magnetic resonance imaging patterns of premenopausal gynaecological conditions among women at Muhimbili National Hospital

**DOI:** 10.1371/journal.pone.0340424

**Published:** 2026-01-21

**Authors:** Merciana G. Mliga, Zuhura Nkrumbih, Ramadhan Kazema, Amina Mbaga

**Affiliations:** 1 Department of Radiology and Imaging, Muhimbili University of Health and Allied Sciences, Dar es Salaam, Tanzania; 2 Department of Radiology and Imaging, Muhimbili National Hospital, Dar es Salaam, Tanzania; Athens Medical Group, Psychiko Clinic, GREECE

## Abstract

**Background:**

Gynecological conditions are common among premenopausal women and significantly affect fertility, quality of life, and productivity. Magnetic Resonance Imaging (MRI) offers superior soft tissue characterization compared to other modalities but local data on MRI patterns in Sub-Saharan Africa remain limited.

**Objective:**

To determine the MRI patterns of gynecological conditions among premenopausal women at Muhimbili National Hospital (MNH), Dar es Salaam, Tanzania.

**Materials and methods:**

A prospective descriptive cross-sectional study was conducted from July to December 2022 among 100 premenopausal women aged 18–45 years referred for pelvic MRI. Data on socio-demographic, clinical, and imaging findings were analyzed using SPSS version 20. Associations were assessed using chi-square and logistic regression, with significance set at p < 0.05.

**Results:**

The majority of participants (80%) were aged above 30 years, and 51% were nulliparous. The prevalence of gynecological conditions was 82%, with uterine fibroids being the most common (37%), followed by simple ovarian cysts (8%) and ovarian tumors (7%). Lower abdominal pain (65%) was the most frequent symptom. Women aged ≥30 years (OR=10.4, 95% CI: 2.5–43.1, p = 0.001) and nulliparous women (OR=5.2, 95% CI: 1.9–13.6, p = 0.001) were significantly more likely to have fibroids, while parity was protective against other gynecological tumors (OR=0.3, 95% CI: 0.1–0.9, p = 0.03). MRI demonstrated characteristic lesion patterns aiding differentiation between benign and malignant lesions.

**Conclusion:**

Gynecological conditions are highly prevalent among premenopausal women, with fibroids being the leading diagnosis. MRI plays a key role in their detection and characterization. Advancing age and nulliparity are significant predictors of fibroids, while parity appears protective against other tumors.

**Recommendations:**

Further multi-center studies incorporating histopathological correlation and longitudinal follow-up are recommended to strengthen diagnostic accuracy and clinical applicability.

## Introduction

Gynecological conditions affect the female reproductive system and are broadly categorized into adnexal, uterine, cervical, and vaginal pathologies. They may be physiological or pathological, and include a wide range of disorders such as infections, neoplastic conditions, vascular conditions, inflammatory disorders, endocrine and metabolic abnormalities, congenital anomalies and trauma-related complications. These conditions disproportionately affect women of reproductive age, leading to significant consequences on fertility, reproductive health, quality of life, and economic productivity, since this age group constitutes the majority of the labor force in many countries [1].

The majority of women presenting with gynecological conditions are premenopausal. Common conditions in this group include pelvic organ prolapse, infertility, reproductive tract infections, and menstrual problems [2]. Symptoms such as lower abdominal pain, abnormal vaginal bleeding, and discharge are frequent and can result in complications such as depression, reduced quality of life, and infertility [3–5]. Among benign conditions, uterine fibroids are the most prevalent, followed by adenomyosis, endometriosis, and polycystic ovarian syndrome, all of which represent a major public health concern [6,7]. Malignant tumors such as cervical, endometrial, and ovarian cancers also remain significant, with global patterns showing rising rates of uterine cancer linked to obesity [8–10]. In addition, congenital anomalies and pelvic inflammatory disease further contribute to infertility [11,12].

Accurate diagnosis of these conditions is essential for timely management. While ultrasound remains the first-line modality due to its availability and affordability, its limitations are well recognized. CT is useful for staging but provides inferior soft tissue contrast compared to MRI. MRI offers superior soft tissue characterization and is the preferred modality for local staging of malignant tumors and for further evaluation when ultrasound is inconclusive [13]. MRI is particularly valuable in characterizing benign tumors, inflammatory disease, and congenital anomalies, as it allows detailed assessment of the extent of disease.

Despite the proven role of MRI, there is limited data from Sub-Saharan Africa, including Tanzania, on the spectrum of gynecological conditions as diagnosed by MRI. Most published studies originate from high-income countries, where disease patterns, healthcare access, and diagnostic resources differ substantially from those in low- and middle-income countries. This knowledge gap makes it difficult to apply global data to local populations.

Therefore, this study aimed to determine the pattern of gynecological conditions among premenopausal women using MRI at Muhimbili National Hospital. By focusing on this specific population, who bear the greatest burden of gynecological morbidity, this study provides context-specific evidence that may improve diagnosis, facilitate early staging, and guide appropriate management, ultimately contributing to reducing the burden of gynecological disease in Tanzania.

## Materials and methods

### Study design

This was a prospective descriptive cross-sectional study conducted from July to December 2022.

### Study setting

The study was conducted at the Radiology Department of Muhimbili National Hospital, the teaching hospital of Muhimbili University of Health and Allied Sciences in Dar es Salaam, Tanzania.

### Study population

All premenopausal women aged between 18–45 years who were referred for pelvic MRI at Muhimbili National Hospital during the study period were considered for recruitment.

### Inclusion criteria

Premenopausal women aged 18–45 years presenting with gynecological symptoms and referred for pelvic MRI.

### Exclusion criteria

Women who were pregnant or declined to provide consent were excluded.

### Sample size determination

The sample size was calculated using the Kish and Leslie formula and adjusted with the finite population correction, yielding a final sample of 100 patients.

### Sampling technique

A consecutive sampling approach was applied. Patients meeting the inclusion criteria were approached and informed about the study. After written informed consent was obtained, eligible participants were enrolled.

### Patient preparation protocol

All patients completed a pre-MRI safety screening form to assess the presence of metallic objects or implanted medical devices. Patients were instructed to have a moderately filled bladder by drinking of water 1 hour before imaging. No fasting was required. Metal objects were removed from the patients.

### MRI technique

All examinations were performed using a 3 Tesla Siemens Magnetom Skyra (Siemens Healthcare, Erlangen, Germany) MRI scanner with a dedicated pelvic phased-array surface coil. Patients were scanned in the supine position.

The standard pelvic MRI protocol included T2-weighted fast spin-echo images acquired in the axial, sagittal, and coronal planes with a slice thickness of 3 mm and an interslice gap of 0.5 mm, as well as T1-weighted spin-echo images in the axial and sagittal planes. Fat-suppressed T1-weighted images were obtained before and after intravenous contrast administration, and diffusion-weighted imaging (DWI) was performed with b-values of 0, 500, and 1000 s/mm^2^, alongside apparent diffusion coefficient (ADC) maps. Typical technical parameters included a field of view (FOV) of 240 mm, a matrix size of 320 × 320, and repetition time (TR) and echo time (TE) values optimized per sequence according to pelvic imaging protocols at 3 Tesla. Intravenous contrast enhancement was achieved using Gadopentetate Dimeglumine at a dose of 0.1 mmol/kg body weight.

### Image quality assessment

Image quality was assessed subjectively by the reporting radiologists using a 3-point scale (good, acceptable, poor). Scans with poor image quality due to motion artifacts or incomplete coverage were excluded. Only scans graded as good or acceptable were included in the final analysis.

### Image interpretation and reader experience

MRI scans were independently reviewed by two certified radiologists with over 5 years experience. To aid accurate image interpretation, the radiologists were provided with patients’ presenting clinical symptoms only. They were blinded to additional clinical information, including laboratory findings and confirmed diagnoses, to reduce potential interpretation bias. In cases of discrepancy, consensus was reached through joint review. Recorded MRI features included lesion location, size, margins, signal intensity, enhancement pattern, presence of metastases, and lymphadenopathy. Final imaging diagnoses were made after consensus.

### Data collection

A structured questionnaire was used to record socio-demographic data (age, parity, marital status, level of education) and clinical details (medical record number, presenting symptoms). Imaging findings were entered into a standardized data collection form based on diagnosis as interpreted on MRI.

### Data analysis

Data was entered, cleaned, and analyzed using SPSS version 20. Results were summarized using frequencies and proportions. Associations between socio-demographic factors and MRI findings were explored using chi-square tests, with a p-value <0.05 considered significant. Logistic regression was used to assess independent associations.

### Ethical considerations

This study was reviewed and approved by the Muhimbili University of Health and Allied Sciences (MUHAS) Institutional Review Board (Approval number: MUHAS-REC-07-2022-1283). Written informed consent was obtained from all participants prior to enrollment in the study.

## Results

A total of 100 premenopausal women who met the inclusion criteria were enrolled in the study. The majority (80%) were aged above 30 years, and 51% were nulliparous Most participants had attained secondary or higher education (86%).

**Table 1** summarizes the socio-demographic characteristics.

**Table 1:** Socio-demographic characteristics among premenopausal women referred for pelvic MRI at MNH from July to December 2022 (N = 100).

Variable	Categories	Frequency (%)
Age	≤30	20(20)
Mean 39.9	>30	80(80)
Marital Status	Single	32(32)
	*Ever Married	68(68)
Parity	Nulliparous	51(51)
	Parous	49(49)
Level of Education	Primary school	14(14)
	Secondary School	47(47)
	University	39(39)

* Ever married includes married, divorced, and widowed participants.

The prevalence of gynecological conditions among premenopausal women was 82%, while 5% had non-gynecological findings and 13% had normal pelvic MRI. The most common condition was uterine fibroids (37%), followed by simple ovarian cysts (8%) and ovarian tumors (7%).

The most frequent clinical presentation was lower abdominal pain (65%), followed by per vaginal bleeding (19.2%).

**Fig 1:**
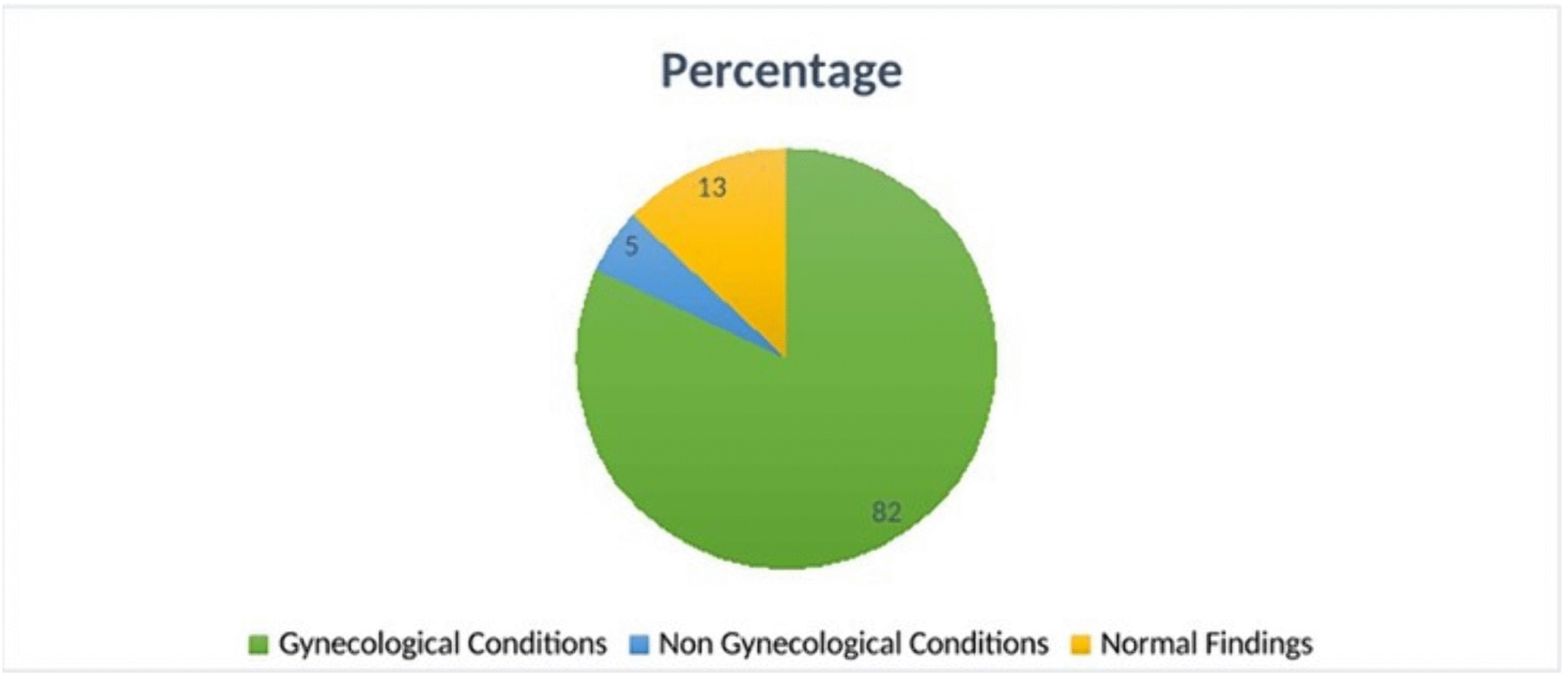
Pie Chart showing prevalence of gynaecological conditions among premenopausal women referred for pelvic MRI at MNH from July to December 2022.

This is as seen in Fig 1, Table 2 and Table 3.

**Table 2:** Frequencies and proportions of conditions among premenopausal women referred for pelvic MRI at MNH from July to December 2022 (N = 100).

Diagnosis	Frequency	Percent
Fibroids	37	37
*Others	9	9
Simple Ovarian Cysts	8	8
Ovarian tumor	7	7
Cervical tumor	6	6
Hydrosalpinx	3	3
Tubo ovarian abscess	2	2
**Non-Gynaecological conditions	5	5
Complex Ovarian cysts	3	3
Adenomyosis	2	2
Vaginal tumor	2	2
Congenital anomaly	2	2
Endometrial tumor	1	1
Normal Findings	13	13
Total	100	100

* Others include: Hematometra, polyp, adenomyosis, lipoma, & pelvic congestion syndrome.

**Non-Gynaecological Conditions: Rectal tumor, bladder tumor and pelvic fracture.

**Table 3:** Clinical presentations among premenopausal women referred for pelvic MRI at MNH from July to December 2022 (N = 120).

Clinical presentations	Frequency	Percent (%)
Lower abdominal pain	78	65
Pelvic mass	13	10.8
PV bleeding	23	19.2
Abdominal distension	2	1.7
PV discharge	4	3.3

*Some patients presented with more than 1 symptom.

**Fig 2:**
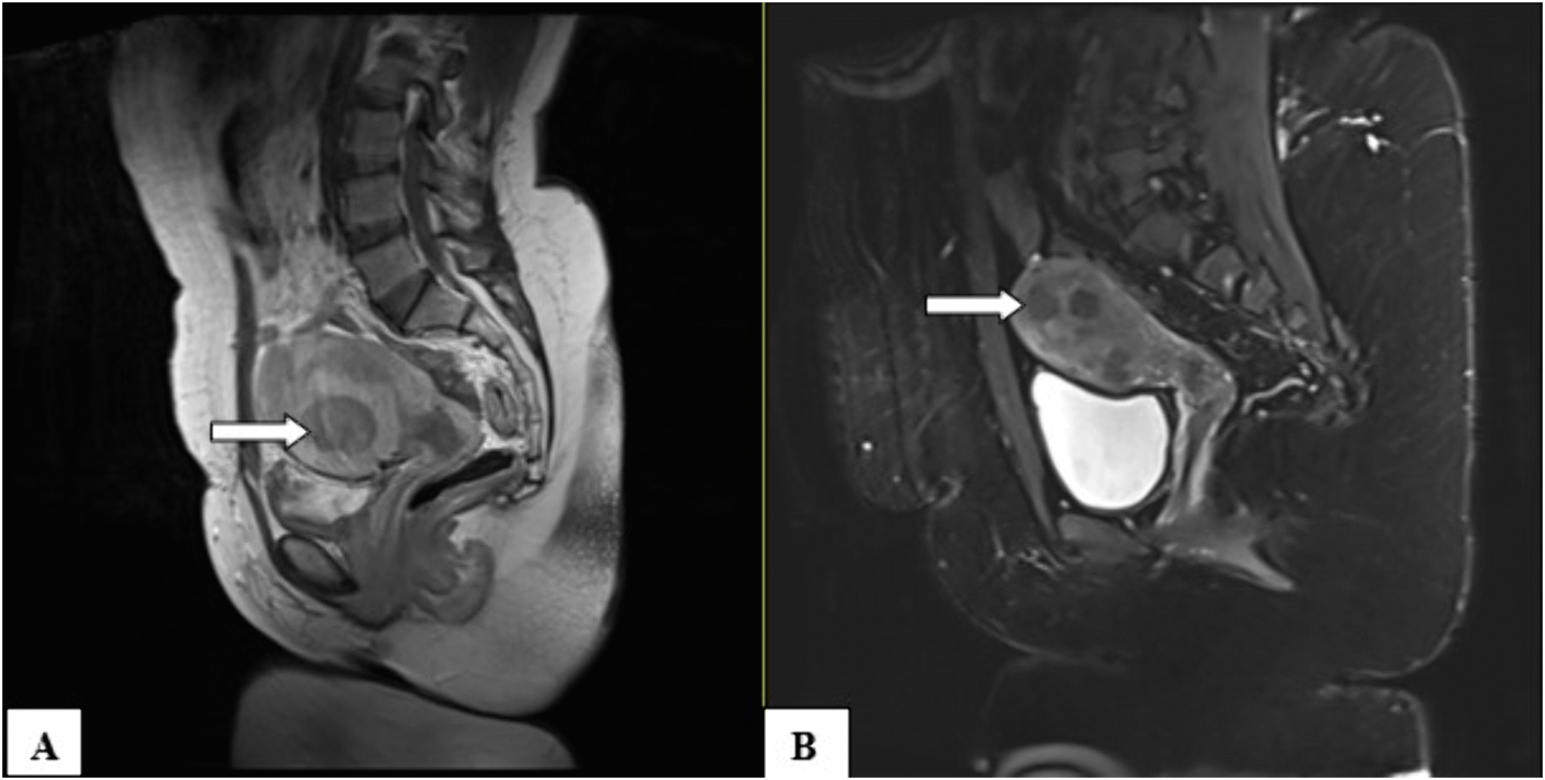
MRI pelvis done at MNH, T2W sequences (Image B with fat suppression) in sagittal plane from 2 different patients aged 35 and 39 years respectively.

**Fig 3:**
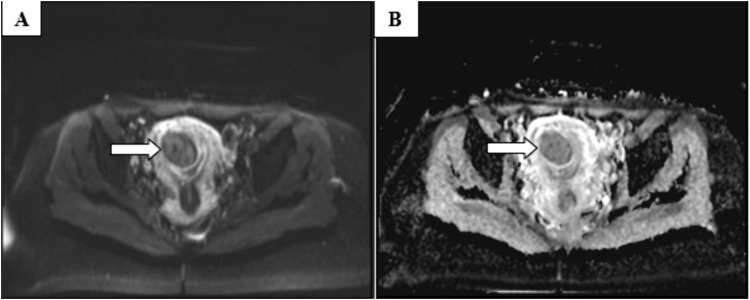
Pelvic MRI of a 35-year-old female performed at MNH, showing DWI and ADC sequences (A &B) respectively.

All 37 fibroid cases had well-defined margins. Most were intramural (45.9%), and 43.3% had multiple lesions. On MRI, fibroids were predominantly hypointense on T1 (94.6%) and T2 (89.2%), with homogeneous enhancement on post-contrast images in 59.5%. Diffusion restriction was seen in 13.5% of cases (Table 4). Fig 2 and 3 show patterns of uterine fibroids as seen on MRI.

**Table 4:** Patterns of Fibroids on pelvic MRI among premenopausal women from July to December 2022 (N = 37).

Pattern		n (%)
Location	Submucosal	2(5.4)
	Intramural	17(45.9)
	Subserosal	1(2.7)
	Submucosal & intramural	5(13.5)
	Subserosal& Intramural	8(21.6)
	Submucosal, Intramural and subserosal	3(8.2)
	Cervical	1(2.7)
Margins	Well defined	37(100)
	Ill defined	–
No. of lesions	1	13(35.1)
	2	8(21.6)
	Multiple	16(43.3)
Consistency	Solid	36(97.3)
	Cystic	–
	Mixed	1(2.7)
T1W	Hypointense	35(94.6)
	Hyperintense	–
	Intermediate	2(5.4)
T2W	Hypointense	33(89.2)
	Hyperintense	2(5.4)
	Intermediate	2(5.4)
Enhancement	Homogenous	22(59.5)
	Heterogenous	14(37.8)
	No enhancement	1(2.7)
DWI/ADC	Restriction	5(13.5)
	No restriction	32(86.5)

Both (A & B) appear with multiple intramural fibroids which are well defined. The fibroids appear with hypointense signals on both images in comparison to the myometrium (White arrows highlight uterine fibroids within the myometrial layer).

The images reveal a well circumscribed intramural uterine fibroid demonstrating no diffusion restriction appearing hypointense on DWI and ADC, consistent with a benign lesion (White arrows indicate uterine fibroids).

Among the 7 ovarian tumors, 57.1% were solid and 71.4% showed heterogeneous enhancement with restricted diffusion (85.7%). Of the 6 cervical tumors, 66.7% had ill-defined margins and restricted diffusion (Table 5).

**Table 5:** Patterns of other gynaecological tumors on MRI among premenopausal women from July to December 2022 (Ovarian tumors N = 7 and Cervical tumors N = 6).

Pattern		Ovarian n (%)	Cervical n (%)
Margins	Well defined	6(85.7)	2(33.3)
	Ill defined	1(14.3)	4(66.7)
No. of lesions	1	6(85.7)	5(83.3)
	2	1(14.3)	1(16.7)
	Multiple	–	–
Consistency	Solid	4(57.1)	5(83.3)
	Cystic	2(28.6)	1(16.7)
	Mixed	1(14.3)	–
T1W	Hypointense	6(85.7)	2(33.3)
	Hyperintense	1(14.3)	–
	Intermediate	–	4(66.7)
T2W	Hypointense	2(28.6)	1(16.7)
	Hyperintense	5(71.4)	3(50)
	Intermediate	–	2(33.3)
Enhancement	Homogenous	2(28.6)	1(16.7)
	Heterogenous	5(71.4)	5(83.3)
	No enhancement	–	–
DWI/ADC	Restriction	6(85.7)	4(66.7)
	No restriction	1(14.3)	2(33.3)
Lymphadenopathy	Present	1(14.3)	5(83.3)
	Absent	6(85.7)	1(16.7)
Metastasis	Present	3(42.1)	4(66.7)
	Absent	4(57.9)	2(33.3)

Adnexal lesions observed on pelvic MRI included ovarian cysts, hydrosalpinx, and tubo-ovarian abscesses. Simple ovarian cysts (N = 8) were mostly right-sided (75%), well-defined, purely cystic, T1 hypointense, T2 hyperintense, and showed no enhancement in 87.5% of cases. Complex ovarian cysts (N = 3) were also predominantly right-sided (66.7%), well-defined, T1 hypointense, T2 hyperintense, with peripheral enhancement, diffusion restriction, and wall thickening or septations in 66.7%. Hydrosalpinx (N = 3) was typically left-sided, T1 hypointense, T2 hyperintense, without enhancement or diffusion restriction. Tubo-ovarian abscesses (N = 2) demonstrated peripheral enhancement and diffusion restriction. These MRI findings are summarized in Table 6, which presents all benign adnexal lesions identified on imaging.

**Table 6:** Patterns of Adnexal lesions on MRI among premenopausal women from July to December 2022 (Simple cysts N = 8, Complex cysts = 3, Hydrosalpinx N = 3, Tubo-ovarian abscess = 2).

Pattern		Simple Cysts n (%)	Complex Cysts n (%)	Hydrosalpinx n (%)	Tubo-ovarian abscess n (%)
Location	Right adnexa	6(75)	2(66.7)	1(33.3)	1(50)
	Left adnexa	2(25)	1(33.3)	2(66.7)	1(50)
Margins	Well defined	8(100)	3(100)	N/A	N/A
	Ill defined	–	–		
No. of lesions	1	8(100)	3(100)	N/A	N/A
	2	–	–		
	Multiple	–	–	N/A	N/A
Consistency	Solid	–	–		
	Cystic	8(100)	3(100)	N/A	N/A
	Mixed	–	–		
T1W	Hypointense	8(100)	3(100)	3(100)	2(100)
	Hyperintense	–	–	–	–
	Intermediate	–	–	–	–
T2W	Hypointense	–	–	–	–
	Hyperintense	8(100)	3(100)	3(100)	2(100)
	Intermediate	–	–	–	–
Enhancement	Homogenous	1(12.5)	–	–	–
	None	7(87.5)	–	3(100)	–
	Peripheral	–	3(100)	–	2(100)
DWI/ADC	Restriction	2(25)	3(100)	3(100)	2(100)
	No restriction	6(75)	–	–	–

*N/A Not applicable.

Age and parity showed significant associations with fibroid occurrence (Table 7).

**Table 7:** Association between socio-demographic characteristics and Fibroids among premenopausal women referred for pelvic MRI from July to December 2022 (N = 100).

Variable	Category	Yes (%)	No (%)	P-Value	OR	95% CI
Age	≤30	3(15)	17(85)	**0.001**	1 (Ref) 10.4	2.5 −43.1
	>30	34(42.5)	46(57.5)			
Parity	Parous	13(26.5)	36(73.5)	**0.001**	1 (Ref) 5.2	1.9-13.6
	Nulliparous	24(47.1)	27(52.9)			
Marital Status	Single	9(28.1)	23(71.9)	0.207		
	Ever married	28(41.2)	40(58.8)			
Level of Education	Primary	5(35.7)	9(64.3)	0.533		
	Secondary	15(31.9)	32(68.1)			
	University	17(43.6)	22(56.4)			

Women aged ≥30 years were 10.4 times more likely to have fibroids compared to younger women (OR = 10.4, 95% CI: 2.5–43.1, *p* = 0.001). Similarly, nulliparous women had a fivefold increased risk compared to parous women (OR = 5.2, 95% CI: 1.9–13.6, *p* = 0.001).

This suggests that advancing age and nulliparity are strong predictors of uterine fibroid occurrence.

Parity was the only variable significantly associated with other pelvic tumors (Table 8).

**Table 8:** Association between socio-demographic characteristics and *other gynaecological tumors (N = 100).

Variable	Category	Yes (%)	No (%)	P-Value	OR	95% CI
Age	≤30	3(15)	17(85)	0.892		
	>30	13(16.2)	67(83.8)			
Parity	Parous	12(24.5)	37(75.5)	**0.03**	0.3 1(Ref)	0.1-0.9
	Nulliparous	4(7.8)	47(92.2)			
Marital Status	Single	3(9.4)	29(90.6)	0.215		
	Ever married	13(19.1)	55(80.9)			
Level of Education	Primary	2(14.3)	12(85.7)	0.962		
	Secondary	8(17)	39(83)			
	University	6(15.4)	33(84.6)			

Parous women had lower odds of developing other gynecological tumors compared to nulliparous women (OR = 0.3, 95% CI: 0.1–0.9, *p* = 0.03), indicating a possible protective effect of childbirth. No statistically significant associations were found with age, marital status, or level of education.

### Discussion

The majority of the female participants in this study were over 30 years. This finding aligns with similar studies conducted in Baghdad, India, Egypt, and Tanzania. Although postmenopausal women were included in most of these studies, the majority of participants still fell within the premenopausal age group [3,14–16].

According to studies conducted in Kenya and Tanzania, the majority of women with gynaecological conditions fell within the age range of 36–49. These findings were similar to our study. The hormonal changes that women experience during this premenopausal period contribute to the higher incidence of gynaecological conditions among them. Notably, fibroids, also being influenced by hormonal factors, were found to be prevalent in most of these women [17,18].

In contrast the mean age of study participants in a study that was conducted in Turkey was 19.7 [19]. Other studies from China, Italy and London came with findings that adolescents mostly below 18 years, were affected with gynecological conditions such as endometriosis [20–22]. Additionally, while endometrial cancer typically affects older women, cases have been documented in women under 30, including some as young as 21 years [23,24]. The difference in age of the participants was due to the difference in population characteristics and gynaecological conditions that were diagnosed in both studies.

The majority of participants in this research were nulliparous. This was due to various factors, such as the presence of gynaecological conditions that may affect fertility. Additionally, this study may have included a significant number of young women who were not yet actively seeking to conceive. Additionally, a nationwide cohort study in U.S.A found that majority of women were nulliparous, with obesity in adolescence being a significant risk factor for lifetime nulliparity [25]. In Sweden, a study involving over 2.8 million women indicated that infertility, often associated with nulliparity, was linked to a higher incidence of ovarian and endometrial cancers [26]. Another study conducted in India, in comparison to our study however, reported a lower proportion of nulliparous women among those seeking medical care, at 25.7% [27].

Also, a study done in Southwest Uganda with a sample size of 319 patients concluded that majority of the women (63.3%) were multiparous [28].

These differences in parity may be influenced by factors such as the study population, geographic location and cultural or societal factors affecting reproductive choices [29].

The high prevalence of gynecological conditions observed in this study is consistent with findings from India and UK, where rates above 70% have been reported [30,31]. However, studies from Egypt and other parts of India have also reported lower prevalence rates despite larger sample sizes, indicating variability across populations [32–34]. Comparable studies from Europe and the United States demonstrate a range of prevalence rates where, a U.S. population-based study reported gynecological morbidity prevalence around 55%, lower than that reported in Indian and Tanzanian cohorts but similar to findings from Egypt [35]. European studies reveal mixed results, with another UK study reporting prevalence near 68% among women undergoing pelvic imaging and Scandinavian data showing lower rates near 50% [36,37]. These differences likely reflect geographic, demographic, and healthcare access factors influencing reported prevalence [38–40].

Fibroids were the most common gynaecological condition among the study participants similar to studies that were conducted in Tanzania, Kenya and USA [17,38,39].

Contrasting findings were seen in a meta-analysis across Middle Eastern countries and found that the most common gynaecological condition was adenomyosis followed by fibroids [6]. Another study conducted in India concluded that menstrual diseases were the most common condition among women of reproductive age [34]. Dysfunctional uterine bleeding was the most common condition among women of reproductive age in a study that had a sample size of 250 women in another study conducted in India [40].

The difference across studies is due to study designs, population characteristics and modalities used for imaging. The other reason with endometrial tumors being least common in this study is due to that it is usually common in older patients, whereas this study included premenopausal women.

Lower abdominal pain being the most common clinical presentation in this study was also a finding in studies that were conducted in India, China and Italy [41–43].Lower abdominal pain is a symptom that often leads a significant number of patients to seek medical attention, as it can be attributed to inflammatory conditions or degenerative conditions like uterine fibroids.

This was in contrast to another study that was conducted across different countries where menorrhagia was the most common clinical presentation [44,45].

Otherwise PV discharge was a common finding in a study that was conducted in Ethiopia as women were diagnosed with cervical cancer [1].

The variation in the nature of common clinical presentations in different studies is explained by the nature of diseases that women were diagnosed with.

In this study, fibroids were identified as the most common finding, and their characteristics were consistent with a previous study conducted in Italy, where the majority of these lesions appeared hypointense on both T1-weighted and T2-weighted MRI sequences [46]. Most fibroids had well-defined borders and homogeneous enhancement, suggesting a low likelihood of invasion into adjacent structures, distinguishing them from malignant conditions such as leiomyosarcoma. Intramural location and multiple lesions were common features, in line with findings from another MRI-based study [47].

Additionally, similar imaging characteristics were reported in a European multicenter study where fibroids predominantly presented as hypointense lesions with well-defined margins and homogeneous post-contrast enhancement on MRI, reinforcing the diagnostic criteria [48]. In USA, a study also noted consistent fibroid imaging patterns, but highlighted that degeneration types varied, including hyaline, cystic, and myxoid changes, influencing signal intensity [49].

The location of fibroids within the uterus can have important fertility implications in women of reproductive age [48,49]. Variations in fibroid imaging patterns assist clinicians in diagnosing the degree of degeneration and guiding treatment planning.

A study on ovarian tumors from China reported that most lesions were solid, in contrast to the mixed consistency commonly observed in this study, although both showed similar signal patterns hypointense on T1-weighted and hyperintense on T2-weighted sequences [50]. Solid lesions in our study frequently demonstrated restricted diffusion, consistent with other studies in China and USA, likely due to the high cellular density of solid tumors [13,51]. Lesion margins also provided important diagnostic information, with most appearing benign, as also seen in a Japanese cohort [52].

Comparable findings have been reported in Europe. A France study of 202 adnexal masses found that malignant tumors typically exhibited high signal on diffusion-weighted imaging (DWI) and low apparent diffusion coefficient (ADC) values, whereas benign lesions had lower or mixed T2 signal intensity and higher ADC values [53]. Similarly, USA research on sex cord-stromal tumors showed that benign lesions often had mild enhancement and low to moderate DWI signal, with malignant lesions showing higher signal intensity and more restricted diffusion [54]. Another study in USA also demonstrated that malignant ovarian tumors tend to have significantly lower ADC values compared to benign ones, supporting the utility of diffusion restriction as a diagnostic marker [55]. Other studies from China and USA described varying MRI patterns in mixed cystic-solid masses, noting that heterogeneous enhancement and restricted diffusion in solid areas were strong indicators of malignancy [50,56]. These findings reinforce the diagnostic role of MRI in characterizing ovarian tumors based on consistency, signal intensity and margin features. This further demonstrates how different MRI sequences contribute to the non-invasive characterization of lesions before pathology is performed.

Metastatic ovarian lesions were less than 50% in this study which is similar to a study that was conducted in China where 15.3% had metastasized at least to the lymph nodes [50]. The slightly higher number of patients with metastatic lesions in this study could be explained by the sample size and nature of the study. The presence of lymph nodes is associated with most lesions being more malignant in nature and staging would help in better management of such patients.

A study conducted in Japan also concurred with this study as most of the cervical tumors appeared with ill-defined margins, hyperintense on T2W and hypointense on T1W. More than 50% of patients with cervical tumours showed adjacent soft tissue invasion, which was noted by the T2W sequence and helped determine adjacent tumour invasion [57]. More than 50% of patients in a study that was conducted in Italy also showed, adjacent soft tissue invasion in cervical cancer patients [58].

The high proportion of invasion reported in both studies and the current findings suggests that many patients tend to present at advanced disease stages. This pattern may reflect delays in diagnosis and limited access to routine screening services in many settings. Consequently, MRI plays an essential role not only in accurate local staging but also in guiding management decisions, particularly for patients presenting late with locally advanced disease.

Based on the patterns of disease in this study, most of the studies had similar findings and this shows that different conditions have different patterns on MRI. The major added advantage of MRI is being with the ability to distinguish adjacent soft tissue easily and be able to better stage a disease prior to any intervention. MRI has an added advantage due to different sequences that helps distinguish lesions based on the appearance. Each sequence has a role to play in coming up with a final diagnosis.T2W has proven to be helpful in better characterization of some of the lesions [59] Variations across different studies was brought about by the nature of the lesions either being malignant or benign in nature.

Being above 30 years old was significantly associated with being diagnosed with fibroids, similar to a study that was conducted in North East Slovenia, Saudi Arabia and Uganda [28,60,61] Variation in estrogen levels across the life span in women is a contributing factor.

Being nulliparous was significantly associated with being diagnosed with fibroids which is a similar finding in studies conducted in India and Italy where multiparous women were even less likely to be diagnosed with fibroids as compared to women who were nulliparous or had at least 1–2 children [43,44]. A study done in Uganda came with different findings where multiparity was associated with having fibroids [28].

Otherwise, the differences across studies are attributed to the difference in geographical locations, hormonal factors and population characteristics. Estrogen hormone plays a great role in women developing fibroids.

Being parous, was significantly associated with reduced risk of having other gynaecological tumors. This is similar to a study that was conducted in Sweden where a study on ovarian tumors concluded that being parous had a reduced risk of being diagnosed with ovarian tumors [59].

The difference in findings among different studies was because different tumors have different risk factors. This is a call to other researchers to work on each gynaecological condition separately especially pertaining to MRI pelvis as the modality of choice, as most studies focused on other modalities such as ultrasound and CT scan. The descriptive MRI patterns reported in this study have potential clinical relevance. Recognition of these patterns will aid clinicians in making informed management decisions, such as distinguishing between benign and potentially concerning findings, guiding follow-up imaging, or selecting patients for conservative versus surgical management. While formal cost-effectiveness analysis was not performed, the use of MRI for accurate characterization may reduce unnecessary procedures and associated costs. Furthermore, our findings are largely consistent with international imaging standards, supporting the generalizability of these patterns to similar clinical settings. Future studies could explore the direct impact of MRI findings on clinical outcomes and resource utilization. The potential sources of bias in this study were selection bias where participants were recruited from a single health facility, possibly skewing the sample toward those who seek medical care more frequently, such as urban, educated, or symptomatic women. This might not reflect the broader population of women with undiagnosed or asymptomatic gynecological conditions.

### Limitations

This was a single centered study which may not reflect the whole population. This was also a cross-sectional study hence the temporal link between the exposures and outcomes was not easily obtained as both were examined at the same time.

## Conclusion

Premenopausal women commonly experience a significant occurrence of gynaecological conditions. These conditions exhibit distinct MRI patterns. Most women above 30 years are affected by gynaecological conditions with lower abdominal pain as the most common clinical presentation and fibroids being the most common condition. Being above 30 years and nulliparous is significantly associated with premenopausal women having fibroids. On the other hand, parity is significantly associated other gynaecological tumors.

### Recommendations

Future research directions should target multi-center prospective studies with larger and more diverse populations to improve generalizability and reduce selection bias. Integration of histopathological data to validate MRI findings and improve diagnostic accuracy, especially for complex adnexal masses.
